# Potentials of Ultrahigh-Field MRI for the Study of Somatosensory Reorganization in Congenital Hemiplegia

**DOI:** 10.1155/2018/8472807

**Published:** 2018-11-25

**Authors:** Simona Fiori, Laura Biagi, Paolo Cecchi, Giovanni Cioni, Elena Beani, Michela Tosetti, Mirco Cosottini, Andrea Guzzetta

**Affiliations:** ^1^Department of Developmental Neuroscience, IRCCS Fondazione Stella Maris, Calambrone, Pisa, Italy; ^2^Laboratory of Medical Physics and Biotechnologies for Magnetic Resonance, IRCCS Fondazione Stella Maris, Calambrone, Pisa, Italy; ^3^Imago7, Pisa, Italy; ^4^Department of Translational Research and New Technologies in Medicine and Surgery, University of Pisa, Italy; ^5^Department of Clinical and Experimental Medicine, University of Pisa, Pisa, Italy

## Abstract

Reorganization of somatosensory function influences the clinical recovery of subjects with congenital unilateral brain lesions. Ultrahigh-field (UHF) functional MRI (fMRI) with the use of a 7 T magnet has the potential to contribute fundamentally to the current knowledge of such plasticity mechanisms. The purpose of this study was to obtain preliminary information on the possible advantages of the study of somatosensory reorganization at UHF fMRI. We enrolled 6 young adults (mean age 25 ± 6 years) with congenital unilateral brain lesions (4 in the left hemisphere and 2 in the right hemisphere; 4 with perilesional motor reorganization and 2 with contralesional motor reorganization) and 7 healthy age-matched controls. Nondominant hand sensory assessment included stereognosis and 2-point discrimination. Task-dependent fMRI was performed to elicit a somatosensory activation by using a safe and quantitative device developed ad hoc to deliver a reproducible gentle tactile stimulus to the distal phalanx of thumb and index fingers. Group analysis was performed in the control group. Individual analyses in the native space were performed with data of hemiplegic subjects. The gentle tactile stimulus showed great accuracy in determining somatosensory cortex activation. Single-subject gentle tactile stimulus showed an S1 activation in the postcentral gyrus and an S2 activation in the inferior parietal insular cortex. A correlation emerged between an index of S1 reorganization (distance between expected and reorganized S1) and sensory deficit (*p* < 0.05) in subjects with hemiplegia, with higher distance related to a more severe sensory deficit. Increase in spatial resolution at 7 T allows a better localization of reorganized tactile function validated by its correlation with clinical measures. Our results support the S1 early-determination hypothesis and support the central role of topography of reorganized S1 compared to a less relevant S1-M1 integration.

## 1. Introduction

Over the last years, the risk of somatosensory impairment in children with unilateral cerebral palsy (CP) has been increasingly recognized, becoming a consistent target for both evaluation and intervention. Studies on tactile dysfunction in unilateral CP report a variable prevalence of deficits ranging from 42 to 90% of children, with stereognosis and two-point discrimination (2PD) as the most frequently impaired aspects [1-4]. A tactile dysfunction has a negative impact on the quality of movements, limits the ability of the child to interact with the environment, and, most importantly, contributes to the progressive functional impairment of the affected upper limb secondary to the so called “learned nonuse” [5-7].

Besides its clinical recognition, there is a growing interest in the understanding of the neuroplastic mechanisms of the somatosensory system after congenital brain lesions, alongside with the better-known reorganization of the corticospinal system. Reorganization of the afferent thalamocortical sensory tracts to primary sensory cortex (S1) has been hypothesized to be related mostly to the capability of ascending fibers to bypass pre- or perinatal lesions and reach the expected cortical destination in the postcentral gyrus [8]. This mechanism seems however imperfect, resulting in some degree of sensory deficit as demonstrated by several studies [9-11].

A significant contribution to the study of brain reorganization of the somatosensory system in unilateral CP was traditionally provided by functional MRI studies, although with significant limitations in spatial resolution. Indeed, studies on sensorimotor reorganization with clinical-field MRI (i.e., 1.5 or 3 tesla) are unable to accurately circumscribe the primary sensory area at a single-subject level, including the distinction, within the perirolandic region, between primary sensory and primary motor activation [12, 13].

The increased availability of ultrahigh-field (UHF) MRI (≥7 tesla) constitutes a unique opportunity for the study of the relationship between structure and function in the human brain, as clearly shown by the first studies in healthy subjects [14]. Compared to lower field MRI, UHF MRI has an increased spatial resolution, with expected increase of sensitivity and specificity of fMRI activation [15]. To date, however, no studies have explored the capability of UHF fMRI in the characterization of brain plasticity in hemiplegic subjects with congenital brain lesions.

We here preliminarily investigated the potentials of UHF fMRI for the study of somatosensory reorganization in adolescents or young adults with congenital hemiplegia. In particular, we aimed to test the following hypotheses: (i) in the affected hemisphere (i.e., the hemisphere contralateral to the hemiparetic side), S1 activation is dislocated from the expected area and (ii) the degree of the dislocation of the reorganized S1 correlates with the severity of the somatosensory deficit.

To test our hypotheses, we performed task-dependent fMRI at 7 T by applying a passive, gentle tactile stimulation in hemiplegic and in control subjects, through an automated 7 T MRI-compatible device developed ad hoc. As the control study, we used a sensory task consisted of passively brushing of fingers by means of a toothbrush [9].

The device showed the capability of a reliable specific activation of a tactile postcentral region at 7 T. The coordinates of the activation obtained in each hemiplegic subject were compared with the expected site of activation (S1 localizer) as defined through a group analysis performed on controls. Finally, in the hemiplegic subjects, the degree of the dislocation of S1 was correlated to the severity of tactile sensory deficits as assessed by stereognosis and 2PD.

## 2. Materials and Methods

### 2.1. Subjects

Ten adolescents or young adults (8 males, mean age of 26 ± 7 years) with congenital hemiplegia (7 right hemiplegic subjects) were recruited for the study. Subjects were selected from a registry of patients with congenital hemiplegia treated at IRCCS Fondazione Stella Maris.

In order to allow for good levels of collaborations during the experiment, only subjects with an IQ above 70 and no reports of psychiatric comorbidities were considered eligible.

Contraindications to MRI were considered as exclusion criteria. Seven right-handed, healthy subjects (3 males, mean age of 29 ± 6 years) were enrolled from the community as controls.

The research project was approved by the Pediatric Ethics Committee of the Tuscany Region (Florence, Italy) and the Italian Ministry of Health and was carried out in accordance with the Code of Ethics of the World Medical Association (Declaration of Helsinki) for experiments involving humans. Written informed consent in accordance with the authorized protocol was obtained from all adult subjects and from parents or guardians for juvenile participants.

### 2.2. Clinical Assessment

Hemiplegic subjects received a detailed clinical assessment, which included motor and sensory evaluations. Sensory function of the nondominant hand was assessed by using 2-point discrimination (2PD) and stereognosis, which are known to have good clinometric properties and clinical utility [6]. 2PD describes the distance (in millimeters) below which two points of touch stimuli within one dermatome cannot be distinguished anymore, with higher values reflecting stronger impairment. Stereognosis describes the percentage of objects correctly identified during manipulation with the nondominant hand, with lower values reflecting stronger impairment [16]. Motor function of the nonhemiplegic hand was assessed by using the Wolf motor function test (WMFT) as previously described, by including a quality dimension and a time dimension [17, 18], and the assisting hand assessment (AHA) [19]. The reorganization of primary motor and primary sensory functions was also assessed by motor and somatosensory evoked potentials (MEP and SEP). A lesion severity score was applied to the 1.5 T datasets of hemiplegic subjects [20] in order to determine the possible impact of brain lesion extension on S1 reorganization and clinical assessment.

### 2.3. Data Acquisition

Data were acquired on a 7 T Discovery MR950 MRI system (GE Healthcare, Milwaukee, WI, USA), equipped with a 2-channel transmit/32-channel receive coil (Nova Medical, Wilmington, MA, USA). Functional images were acquired to accurately identify S1 at a single-subject level and, only for the control group, to identify the expected site of activation (S1 localizer) at a group level. We used a T2∗-weighted gradient echo (GRE) echo-planar imaging (EPI) sequence (TR = 2000 ms, TE = 21.5 ms, flip angle = 60°, field of view (FOV) = 192 mm × 192 mm, matrix = 128 × 128, isotropic voxel = 1.5 × 1.5 × 1.5 mm^3^). Thirty-two slices (about 5 cm coverage) were placed in order to cover primary and secondary sensory cortices. Each functional series was composed by 160 time points (volumes) and 5 additional initial dummy scans, for a total acquisition time of 5′30^″^. A whole-brain GRE-EPI sequence was acquired with the same parameters of functional series, except for a longer TR (6000 ms), allowing the complete coverage of the brain. One single volume of 90 slices was acquired after 3 dummy scans in 24 seconds. We acquired also a 3D FSPGR T1-weighted sequence (TR/TE = 5.9/2.1 ms; flip angle 12°, isotropic voxel = 1 × 1 × 1 mm^3^, acquisition time = 4′50^″^). Structural images were also acquired, for spatial coregistration of functional 7 T data with anatomical 1.5 T images.

Subjects were also assessed on a 1.5 T Signa HDxt (GE Healthcare, Milwaukee, WI, USA) MR scanner equipped with an 8-channel array coil (Invivo Corporation, Gainesville, FL, USA). In particular, three-dimensional structural images were acquired using a 3D FSPGR T1-weighted sequence (time of repetition (TR)/time of echo (TE) = 11.9/3.6 ms; flip angle 10°, isotropic voxel = 1 × 1 × 1 mm^3^). Since 3D T1-weighted whole brain images at UHF may present signal inhomogeneities that could fail brain segmentation, we used structural images acquired at lower magnetic field for brain segmentation, as well as for the normalization of single-subject brains of control groups in a common space [21].

### 2.4. Functional Tasks

Two different sensory tasks were performed. The first sensory task consisted of a gentle tactile stimulation delivered on the thumb and the index finger of each hand separately, by applying a tactile stimulator developed ad hoc (Linari Engineering, Pisa, Italy). In detail, the tactile stimulator consisted of an MRI-compatible pneumatic system with two pumps (range of pressure = 0 ÷ 0.2 MPa). The pumps were located in the scanner engine room led by a unique remote control in the console room. Each pump was connected to a little vesicle through plastic pipes, filled with air. Each vesicle was applied to the distal phalanx of the index (F1) or thumb (F2) finger. Pumps had been set with a pressure of 0.1 MPa and an inflation/deflation rate of 1 Hz, so as to have about one “touch” every second.

A second sensory task was used as the control study, to compare the gentle tactile to standard task, commonly used in such patients and in clinics [9, 22]. This task consisted of passively brushing hand index and thumb fingers (F1 and F2, respectively), by means of a toothbrush, at a frequency of about 1 Hz.

The experiment consisted of 4 functional series, 2 for each task performed on either hand. fMRI series were built using a block design format (block duration = 20 s), alternating the sensory stimulation to rest according to the following: F1 → rest → F2 → rest. This scheme was repeated 4 times for each sensory stimulus. To the purposes of this paper, a comprehensive activation of F1 and F2 stimulations was considered for the data analysis (see below).

Subjects were asked to keep their eyes closed during rest or stimulus delivery; occlusive earplugs attenuated ambient scanner noise throughout rest and activation periods. Before each functional series, a brief test of task was performed in each subject to have confirmation of sensory stimulus delivery; as well at the end of each series, subjects were asked to confirm the stimulus perception.

### 2.5. Data Analysis

Data analysis was performed with BrainVoyager (Brain Innovation, Maastricht, The Netherlands), by using ad hoc scripts written in MATLAB (MathWorks, Natick, MA, USA). First, each functional series was visually inspected, looking for motion spikes or heavy movement periods. Functional data preprocessing included mean intensity adjustment to compensate for interscan intensity differences, temporal interpolation, and resampling to compensate for slice-dependent time differences (sinc function), 3D motion correction (rigid body transformation, sinc interpolation), and high-pass temporal filtering (GLM-Fourier approach, two cycles).

The 1.5 T 3D T1-weighted images were transformed into the AC-PC coordinate system by applying a six-parameter rigid transformation and turned into Talairach's space. In hemiplegic patients, transformations were calculated on a half-artificial brain, by replacing the lesioned hemisphere with the healthy one, flipped on the sagittal plane.

Functional data were coregistered to the “whole brain” GRE-EPI dataset by using a rigid body alignment, considering that EPI acquisition induces same distortions on images. Moreover, in order to coregister the whole-brain GRE-EPI data to the 7 T structural images, an affine transformation (9 parameters; 3 for translation, 3 for rotation, and 3 for FOV scaling) was automatically calculated, visually inspected and manually corrected by two experienced raters (LB and PC). Finally, a rigid body transformation was calculated to align the 7 T structural images to the analogous acquired at 1.5 T.

In order to preserve UHF spatial resolution, in hemiplegic subjects, the analyses were conducted in the native space, using the inverses of above transformations or rather aligning anatomical images to functional ones, keeping these unvaried. Similar approach was used for 3D visualization of reconstructed surface representation (mesh); 1.5 T T1-weighted images in ACPC space were automatically segmented in order to obtain segmented cortical boundary. The inverses of above spatial transformations were applied to the segmented volumes, to import the segmentation in the space of functional data. Finally, manual correction was used to edit little imperfections.

In controls, spatial transformations were applied to functional images, in order to perform group analysis in a common space.

Blood oxygenation level-dependent (BOLD) responses were analyzed using a general linear model (GLM) approach, modelling the regressors of interest (by convolving a boxcar function for each stimulation block with two gamma functions for the hemodynamic response) and six spurious movement regressors (outputs of the 3D motion correction procedure). The contrasted activity for gentle tactile stimulation of both fingers versus the rest condition ((F1 + F2) > rest) was used to investigate the reorganization of somatosensory cortices.

The same contrast for “brush” stimulation of both fingers versus the rest condition ((F1 + F2) > rest) was used as the control test, to compare results.

First-level statistical analyses were performed using a threshold at *p* < 0.005 (*t* > 2.85) and cluster size > 10 mm^3^, to generate individual subject's maps in native space. With respect to the tactile stimulation, for each hemisphere (contralateral and ipsilateral to the stimulated hand), two specific regions of interest (ROIs) were considered (S1 and S2). By applying the spatial transformations previously described, in order to compare individual variability of activation, the ROIs of each hemiplegic subject were transferred into Talairach's space and the individual center of mass calculated. In controls, following spatial normalization, the coregistered functional datasets were used for a second-level multisubject analysis, by using a fixed-effect (FFX) GLM-based analysis and a statistical threshold corrected for false discovery rate (FDR) *q* < 0.05 corresponding to a *p* < 0.001. For control group activation, the center of mass of each ROI was calculated. In patients, the vector between the expected and single-subject S1 center of mass was determined, as the measure of reorganized S1 dislocation and its length (in millimeters) was assumed as a “dislocation index.” A dislocation index was calculated as well for the activation elicited by the gentle tactile stimulus of the preserved hand in hemiplegic subjects. Standard deviations (Δ*x*, Δ*y*, Δ*z*) of the expected center-of-mass coordinates were used to calculate the radius, *r*_*CG*_, of a sphere describing the expected activation area, according to the following:

(1) $$\begin{array}{c} r_{CG}=1.5\times \sqrt{{\Delta_{x}}^{2}+{\Delta_{y}}^{2}+{\Delta_{z}}^{2}}. \end{array}$$

A paired *t*-test was performed to assess differences in the mean dislocation indices of the dominant and nondominant hand-related activation.

S1 dislocation index was related to sensory deficit assessed by 2PD and stereognosis and to severity of the lesion in the hemisphere contralateral to the nondominant hand by using a one-tailed Pearson correlation index.

## 3. Results

Of the ten enrolled subjects with hemiplegia, two refused to perform 7 T MRI after performing 1.5 T MRI and withdrew from the study without providing explanations, as allowed by the consent agreement. Two further datasets obtained at 7 T were excluded from the following analysis because of the presence of excessive movement artifacts during functional acquisition, which failed the post hoc correction process. Data from six subjects (4 with right hemiplegia and 2 with left hemiplegia, mean age 25 ± 6 years, range = 19 ÷ 36 years) were thus available for analysis.

### 3.1. Clinical Assessment

Clinical characteristics of the six subjects are reported in Table 1, including clinical sensory and motor characteristics and somatosensory and motor reorganization assessed by evoked potentials.

**Figure 1:**
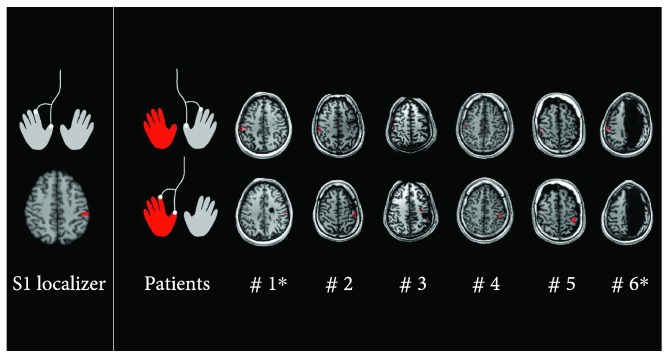
S1 activation rendered on T1 axial images for control group analysis (S1 localizer) and single-subject analysis (patients). The gentle tactile stimulation in the dominant hand elicits a contralateral S1 activation in the group analysis (S1 localizer). Single-subject analyses reveal that the gentle tactile stimulation elicits a unilateral S1 contralateral activation pattern for both nondominant (red) and dominant (grey) hands (patients). ^∗^Right brain lesion.

According to the timing of lesion [23], structural MRI showed brain maldevelopment in one subject (unilateral extensive polymicrogyria with an interhemispheric cyst), periventricular white matter lesion in one subject (i.e., focal venous infarction), cortical and deep grey matter lesions in 3 subjects (focal stroke, <28 days of life), and early acquired brain injury in one subject (focal stroke, around 3rd month of life). All hemiplegic subjects but one had pure unilateral brain lesions. The only subject with bilateral lesions (patient 5, Table 1) had a watershed infarction with very mild white matter abnormalities in the hemisphere ipsilateral to the dominant hand. Despite the presence of focal lesions, anatomical landmark for hand sensorimotor areas (“hand knob”) was successfully identified bilaterally in all subjects (Figure 1) but one (patient 6), the one with extensive polymicrogyria. Brain lesion severity scores [20] are reported in Table 1.

### 3.2. Identification of Primary and Secondary Somatosensory Areas in Control Subjects

The gentle tactile stimulation of dominant hand fingers in controls determined a monolateral activation in the left postcentral gyrus (Broadmann area (BA) 3-1), located at the Talairach's coordinates [*x*, *y*, *z*] = −57 ± 5, −17 ± 3, 44 ± 5, and represented in the left column of Figure 1. This area was identified as the “expected S1 area,” and the dislocation index of each hemiplegic subject was calculated according to its center of mass. Group analysis showed also bilateral activation in the inferior parietal lobule (BA 40-2, averaged coordinates = ±53 ± 3, −25 ± 3, 35 ± 7), classified as S2 areas, and monolateral activation in the right precentral gyrus (BA 6).

### 3.3. Identification of Primary and Secondary Somatosensory Areas in Hemiplegic Subjects

In hemiplegic subjects, fMRI activation at 7 T was carefully checked by three experienced raters (MC, LB, and SF). Thanks to the gentle tactile stimulus, S1 activation foci were successfully mapped in the native space of each subject (Figure 1). ROI coordinates, transformed into Talairach's space, are reported in Table 2. For all hemiplegic subjects, the activation of S1 was clearly unilateral in the hemisphere contralateral to the stimulated hand.

**Figure 2:**
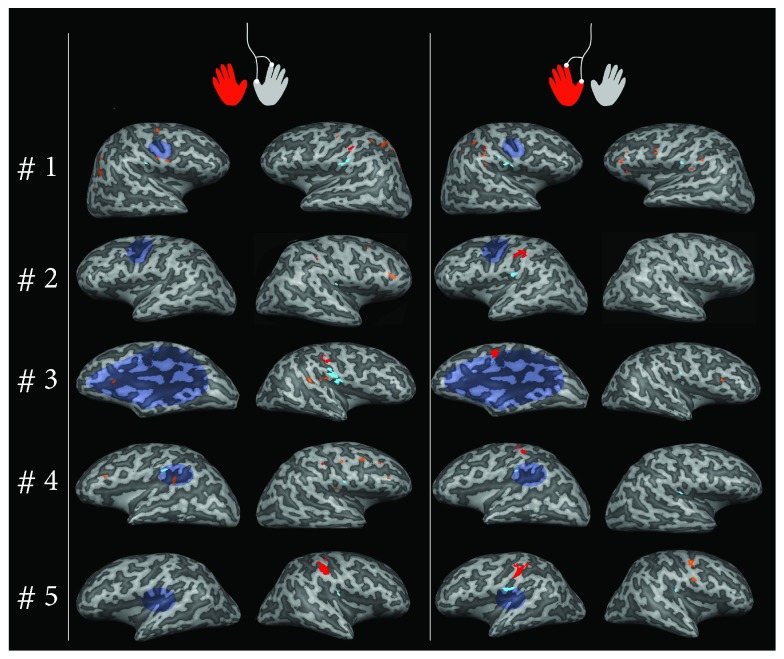
Activation foci for tactile stimulation of both hands, represented on inflated cortices in the native space of each single subject. Subjects' identifiers are shown in the left column, accordingly to Tables 1 and 2. For subject #6, segmentation failed due to the presence of extensive polymicrogyria in lesioned hemisphere, so the representation is missing in this figure. For each subject, the lesion is represented in colored blue transparency on the inflated cortex, approximately corresponding to its anatomical projection on brain surface. S1 responses to tactile stimulation are represented in red, while S2 responses are represented in cyan. Further activation in addition to S1 and S2 for the stimulation of the dominant hand was the following (middle column): #1 HH: PrCG BA 4, IPL BA 40, SPL BA 7, SOG BA 19; LH: PrCG BA 4 and BA 6, Pcu BA 19, MTG BA 39, MOG BA 19. #2 HH: MFG BA 6, IFG BA 46; LH: none. #3 HH: STG BA 22, IPL BA 40; LH: IFG BA 45. #4 HH: PrCG BA 4, MFG BA 6, MFG BA 9, STG BA 22; LH: IPL BA 40, MFG BA 9. #5 HH: none; LH: none. #6 (not represented) HH: IPL BA 40, STG BA 22; LH: none. For the stimulation of the nondominant hand (right column): #1 HH: PrCG BA 6, MFG BA 9-46, IFG BA 46, STG BA,22; LH: IPL BA 40. #2 HH: none; LH: none. #3 HH: IFG BA 44; LH: none. #4 HH: none; LH: none. #5 HH: PrCG BA 4, PrCG BA 6; LH: none. #6 (not represented) HH: IPL BA 40, PrCG BA 6; LH: IPL BA 40, STG BA 22. Abbreviations: HH: healthy hemisphere; LH: lesioned hemisphere; BA: Broadmann area; PrCG: precentral gyrus; MFG: middle frontal gyrus; IFG: inferior frontal gyrus; IPL: inferior parietal lobule; SPL: superior parietal lobule; Pcu: precuneus; SOG: superior occipital gyrus; MOG: middle occipital gyrus; STG: superior temporal gyrus; MTG: middle temporal gyrus.

Activation in the inferior parietal lobule was detected bilaterally in 4 out of 6 patients, while was detected ipsilaterally or controlaterally in one subject each (Table 2 and Figure 2). Further foci of activation in each subject for gentle tactile task are detailed in Figure 2.

Brushing stimulus elicited a similar activity pattern, except for the activation of ipsilateral S1 and medial frontal gyrus (BA 6) (Figure 1 in Supplementary Materials). Indeed, brushing determined huge activation blobs with no anatomical separation between S1 and S2, neither at FDR nor at Bonferroni multiple comparison correction.

### 3.4. Dislocation of the Activation in the Hemiplegic Subjects

**Figure 3:**
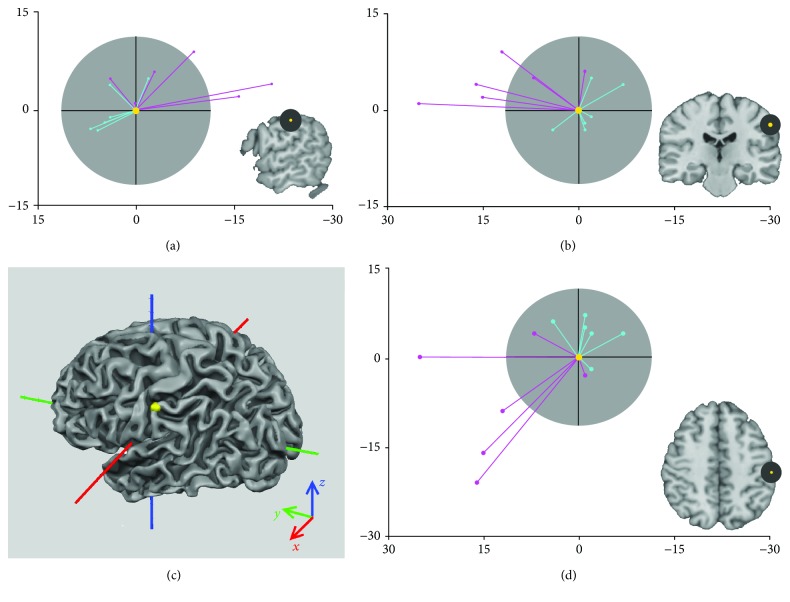
S1 dislocation vectors of each patient for the gentle tactile stimulation of both nondominant (pink vectors) and dominant (cyan vectors) hands, projected in sagittal (a), coronal (b), and axial (d) planes. The yellow point represents the position of S1 resulting from the group analysis (*x* = −57, *y* = −17, *z* = 44) and is set to zero. The sphere of expected localization (<1.5 standard deviation (SD)) in control brains (grey) has radius *r*_*CG*_ = 11.5 mm. Color code of axis respects the convention [*x*, *y*, *z*] → RGB (*x* = red, *y* = green, *z* = blue). Axis scales are in millimeters. In the bottom-left corner (c), the three dimensional representation of S1 control group ROI (yellow) is overlapped on the mesh of the white/grey matter boundary of a standard brain (Colin27 brain, [24]). All dominant hand vectors are within a 1.5 SD, as well as 2 out of 6 hemiplegic subjects. Not the posterior-mesial gradient for S1 dislocation.

**Figure 4:**
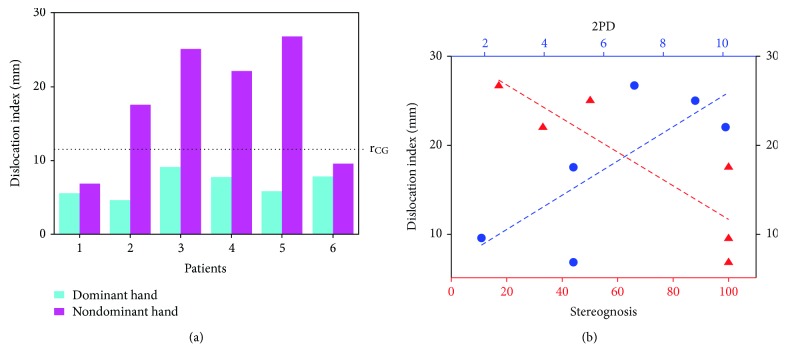
(a) Dislocation indices for nondominant (pink bars) and dominant (cyan bars) hands. All dominant hand S1 dislocation indices are inferior to the radius, *r*_*CG*_ (expected localization according to the control group analysis) as well as 2 out of 6 nondominant hand indices. The latter are of the 2 subjects with no sensory deficit (see Table 1 for clinical details). (b) Correlation between dislocation index and sensory deficit assessed at 2PD and stereognosis. A significant correlation emerged between dislocation index and sensory deficit, with a greater distance being associated to a worse sensory deficit. Abbreviation: 2PD: 2-point discrimination.

The S1 dislocation vector for each hemiplegic subject and its position within the sphere of radius *r*_*CG*_ = 11.5 mm, describing the expected activation, were presented in Figure 3. For the nonhemiplegic hand stimulation, the sphere included the S1 dislocation vector of all patients, while for the hemiplegic hand, the sphere included the S1 dislocation vector of only two of them. S1 dislocation indices of both hands were reported for each single hemiplegic subject in Figure 4(a). Mean dislocation indices of the nondominant hand and the dominant hand in hemiplegic subjects resulted significantly different (*p* < 0.05).

### 3.5. Correlation with Clinical Measures

A significant correlation emerged between reorganized S1 dislocation index and sensory measures, both at stereognosis (*p* = 0.012, *R* = −0.868) and 2PD (*p* = 0.041, *R* = 0.756). In particular, a bigger distance between actual and expected S1 correlated with a more severe tactile deficit (Figure 4(b)).

## 4. Discussion

This is the first study that uses UHF fMRI for the study of somatosensory reorganization in congenital hemiplegia, by applying a reliable gentle tactile stimulus. We were able to confirm our initial hypotheses. Firstly, our results showed a greater S1 variability in the hemisphere contralateral to the hemiparetic side than in the ipsilateral. Secondly, the degree of dislocation of the reorganized S1 correlated with the severity of tactile deficit.

As hypothesized, S1 activation was identifiable in the native space of each hemiplegic subject; neither normalization nor smoothing was applied for functional images in each single-subject analysis, in order to maximize the potential for spatial localization obtained at UHF fMRI. Indeed, our data have a voxel size of about 3.4 mm^3^ compared to previous studies where resolution ranged from 27 to 36 mm^3^ [13, 12]. Somatosensory activation due to the gentle tactile stimulus activated a small area (mean activation among subject ~ 0.7 cm^3^) that was anatomically identified as S1 in the postcentral gyrus in the hemisphere contralateral to the gentle tactile stimulus of the two hand fingers. This active area found in somatosensory cortex has similar characteristics, in terms of location and dimension, to activities detected in precedent investigations of finger somatotopy at 7 T [14, 25, 26]. Both in control and hemiplegic subjects (in the latter case, S1 will be referred to as “reorganized S1” when elicited in the hemisphere contralateral to the nondominant hand), this activation was clearly unilateral. On the contrary, the activation determined by brushing presented a bilateral pattern in two out of six patients and in four out of seven controls (control group analysis in Figure 1 in Supplementary Materials). Moreover, brushing stimulus elicited larger areas (mean activation ~ 3.2 cm^3^), making the segregation of S1 from S2 harder as well as the distinction between S1 (postcentral gyrus activation) and M1 (precentral). These discrepancies can be attributed in part to the higher specificity of the gentle tactile stimulus, in part to physiological differences in the cortical processing of the two stimuli. A further area was clearly identified with a variable pattern (ipsilateral, bilateral, or contralateral to the stimulation side) in the posterior parietal insular cortex, referred to as S2. The activation due to the brushing determined a similar variable pattern, with larger activation.

Recent studies on somatotopy of healthy subjects at 7 T [25, 14, 27-29] employed some form of mechanical and/or electrical stimulation, difficult to apply in the clinical setting. Human touch was also used as stimulus in a 7 T fMRI study to investigate cortical representation of individual fingers [26], with the limitation of reproducibility. The device that we applied in the current study has the advantage of an automated, predetermined, and reproducible stimulation, which is administered to the subject with no collaboration required (with the exception of the general compliance to an MRI exam). This is particularly useful in hemiplegic subjects in which motor deficit, musculoskeletal constraints, or mirror movement can negatively impact on motor activation and image quality in fMRI. Furthermore, by requiring no collaboration by the subject, the device can be potentially applied also to younger ages or, theoretically, to sleeping subjects. Our results thus support the utility of the device for the fMRI study of somatosensory reorganization.

Although limited to the hemisphere contralateral to the hemiplegic side, reorganized S1 showed a certain degree of variability, as previously demonstrated at lower field MRI [13]. Our and previous findings support the hypothesis that interhemispheric reorganization of the primary somatosensory area (S1) is uncommon in congenital brain lesions [13, 8]. Separately and differently from S1, S2 showed a varied pattern of activation, with pure ipsilesional S2 activation found in 1 subject and pure contralesional S2 activation found in 1 subject, while all the remaining subjects had a bilateral S2 activation. These results agree with previous studies, which hypothesized a broader pattern of S2 localization [13, 12].

In order to check if certain grey matter plasticity was possible for S1, despite limited to the lesioned hemisphere, we tried to give the measure of S1 dislocation, by using an expected spherical volume into which allocate the normal probability to have S1 in healthy subjects (radius, *r*_*CG*_). For reorganized S1, we found that only 2 subjects fall into *r*_*CG*_, which is the probability of being in the expected S1. Conversely, in the hemisphere ipsilateral to the nondominant side, S1 always resulted within an *r*_*CG*_ radius. In the previous fMRI study, Wilke et al. [13] compared the topography of S1 in a group of subjects with congenital hemiplegia. They demonstrated a greater variability of S1 location around the central sulcus in the lesioned hemisphere compared to the hemisphere contralateral to the preserved hand. Compared to our results, they did not include any quantitative measure of S1 dislocation nor their findings were validated by clinical measures. Furthermore, our findings add to the previous literature in that a posterior-mesial pattern for somatosensory reorganized function can be identified (see Figure 3).

Due to the higher spatial resolution and accuracy of S1 mapping at 7 T fMRI, our results measured a relevant distance defining a sphere of 2 cm^3^ volume as the expected area to relocate reorganized S1. Due to this marked extension, we may thus assume that a certain degree of cortical plasticity has occurred out of a predetermined somatosensory area in at least 4 out of 6 hemiplegic subjects in our sample, limited to the ipsilesional hemisphere.

Although some degree of cortical plasticity can occur, our results also support previous hypotheses on early determination of S1 [8], if some somatosensory predetermined tissue is preserved. Conversely to Juenger et al. [8], our sample included two earlier occurred lesions, which showed the better tactile clinical profile (patients 1 and 6). Interestingly, one of these two subjects had a very extended polymicrogyria (patient 6) on the side contralateral to the hemiparesis, with a very small amount of apparently preserved cortical grey matter on structural images. However, in that small amount of available normal cortical tissue, it was able to accommodate somatosensory tactile mapping, thus reinforcing the concept of highly defined determination of that area, allowing no sensory deficit on the nondominant hand [30].

A significant correlation emerged in our study between S1 dislocation index and the two measures we used to assess somatosensory deficit. It has been reported in studies on congenital unilateral brain lesions that motor function, especially fine motor control, is worse when M1 reorganization is ipsilateral to the hemiplegic side, likely due to the segregation of motor and somatosensory areas in two different hemispheres [31, 9, 11]. In our sample, we have two subjects with contralateral motor reorganization assessed with MEPs. Interestingly, those subjects have the closest distance to the expected S1 and have better sensory function, with no substantial differences in motor function compared to the group with ipsilesional reorganization. If this observation will be confirmed in a bigger sample of subjects, it would support the hypothesis that the functionality of reorganized S1 depends more on the distance from the expected somatosensory region of the cortex than the distance from M1 [13]. Previous findings and ours add to the previous literature, in the sense that a clinical meaning is given to S1 early determination. It needs to be noticed that, as expected, all subjects with contralateral reorganization have perinatal arterial ischemic lesion. This has been already hypothesized to play a role in limiting white matter ascending thalamocortical afferents to S1 [32]. Finally, as we might have expected a possible impact of brain lesion size on S1 reorganization, we assessed brain lesion severity by using a recently developed semiquantitative system [20]. Interestingly, no relationship emerged between the dislocation index and brain lesion severity. Studies on a larger sample with quantitative measurements of lesion volume will confirm this finding.

In order to further support the relevance of sensory deficit for motor outcome in our sample, we explored at post hoc the relationship between sensory deficit and motor impairment. In particular, we found a correlation between stereognosis and quality dimension at WMFT (*p* = 0.015, *p* = 0.856) and AHA (*p* = 0.045, *p* = 0.753) and between 2PD and quality dimension at WMFT (*p* = 0.036, *p* = −0.773) and AHA (*p* = 0.038, *p* = −0.765). Our results further support a possible influence of tactile deficit on motor control.

The principal limitation of this study is the sample size. The number of subjects included in this sample was limited due to the low prevalence of the disease and the psychological and physical compliance required by the MRI exam; also, two subjects were excluded for excessive movement artifacts, which may be an issue at UHF MRI. However, it has been suggested to consider cautiously but positively, significant results in small cohorts [33]. The small number of subjects does not allow for including the hemiplegic side and sex as a covariate in the analysis. Similarly, due to the limited number of subjects in the final dataset, the potential effect of motor reorganization has not been systematically considered in the analyses but only speculated in the discussion based on single-subject findings. Finally, no comparison between S1 and M1 reorganization was conducted, which might be influenced differently by specific factors.

## 5. Conclusions

For the first time, our study uses UHF fMRI for the study of somatosensory reorganization in congenital hemiplegia, by applying a reliable gentle tactile stimulus. Since the use of UHF MRI is still limited, it is of utmost importance to identify the possible fields for its application, i.e., to define the added value of UHF MRI as compared to conventional MRI. In fact, the increased signal sensitivity at 7 T allows obtaining more reliable BOLD signals in single subjects, compared to lower field strengths, also shortening acquisition duration and block repetitions [25], which is highly recommended in the clinical and pediatric setting. We believe that this initial demonstration of the potentials of UHF in studying adaptive brain plasticity in young adults might foster further research in larger samples of subjects with congenital brain damage and at younger ages.

## Figures and Tables

**Table 1:** Characteristics of subjects with hemiplegia.

Patient	Age	Sex	Side of lesion	2PD	Stereognosis	MEP	SEP	WMFT quality	WMFT time^∗^	AHA	Lesion severity^#^	Lesion type
1	21	M	R	5	100	C	I	4.13	2.27	67	5	II
2	36	M	L	5	100	I	I	5	1.05	89	8.5	IV
3	19	M	L	9	50	I	I	3.67	5.08	70	14	III
4	20	M	L	10	33	I	I	1.47	2.61	38	10	III
5	28	M	L	7	17	I	I	2.67	2.18	59	4	III
6	26	M	R	2	100	C	I	4.53	1.59	84	18.5	I

Abbreviations: 2PD: 2-point discrimination; MEP: motor evoked potentials; SEP: somatosensory evoked potentials; DI: dislocation index; AHA: assisting hand assessment; M: male; L: left; R: right; C: contralesional; I: ipsilesional. ^∗^Expressed in sec. ^#^Out of 40 [20].

**Table 2:** Centre of mass localization, extension, and peak *Z*-score (*Z*∗) for primary (S1) and secondary (S2) somatosensory areas, identified by the gentle tactile stimulation of the paretic hand for each single subject.

Area	Sub	Side	Talairach's coordinates			Cluster size (mm^3^)	Peak's *Z*-score
			*x*	*y*	*z*		
S1	1	c	58 ± 2	−20 ± 3	38 ± 5	439	3.72
	2	c	−45 ± 3	−26 ± 6	53 ± 5	1251	6.31
	3	c	−32 ± 3	−17 ± 4	45 ± 4	675	5.80
	4	c	−42 ± 2	−33 ± 2	46 ± 1	87	3.30
	5	c	−41 ± 5	−38 ± 5	48 ± 4	1595	6.16
	6	c	50 ± 3	−13 ± 1	49 ± 3	174	4.68

S2	1	c	56 ± 5	−16 ± 4	24 ± 2	785	5.56
		i	−57 ± 3	−37 ± 4	18 ± 4	381	3.48
	2	c	−47 ± 3	−19 ± 2	19 ± 3	347	4.90
	3	i	50 ± 3	−16 ± 3	39 ± 1	119	3.89
	4	c	−50 ± 3	−25 ± 2	25 ± 1	223	3.83
		i	53 ± 5	−32 ± 2	19 ± 2	339	3.72
	5	c	−49 ± 3	−24 ± 2	20 ± 3	501	5.34
		i	51 ± 3	−24 ± 2	12 ± 2	152	4.22
	6	c	58 ± 3	−11 ± 6	22 ± 7	1390	6.16
		i	−64 ± 2	−7 ± 3	4 ± 1	151	6.54

Talairach's coordinates are provided as the value and standard deviation, based on all voxels of the region of interest. Abbreviations: c: contralateral to the stimulated hand; i: ipsilateral to the stimulated hand.

## Data Availability

All the data used to support the findings of this study are included within the article.

## Supplementary Materials

Supplementary Materials Figure 1 represents the activation in the primary somatosensory area elicited by the brushing task, for control group analysis (group analysis) and single-subject analysis (patients). The brushing stimulation in the dominant hand elicits a bilateral S1 activation in the group analysis of controls (S1 localizer). Bilateral representation of S1 was found also in single-subject analysis carried on patients. In particular, 2 out of 6 patients had bilateral activation for the brushing stimulation of the dominant hand (grey, top row, patients #1 and #5) and the nondominant hand (red, bottom row, patients #1 and #4). ^∗^Right brain lesion.
