# Supplementary material for: Potentials of Ultrahigh-Field MRI for the Study of Somatosensory Reorganization in Congenital Hemiplegia
Source: Neural Plast. 2018 Nov 25;2018:8472807. doi: 10.1155/2018/8472807 (PMC6286762; doi:10.1155/2018/8472807)
Supplement: Supplementary Materials — Figure 1 represents the activation in the primary somatosensory area elicited by the brushing task, for control group analysis (group analysis) and single-subject analysis (patients). The brushing stimulation in the dominant hand elicits a bilateral S1 activation in the group analysis of controls (S1 localizer). Bilateral representation of S1 was found also in single-subject analysis carried on patients. In particular, 2 out of 6 patients had bilateral activation for the brushing stimulation of the dominant hand (grey, top row, patients #1 and #5) and the nondominant hand (red, bottom row, patients #1 and #4). ∗Right brain lesion. [file 8472807.f1.docx]

**Supplementary Material**

**
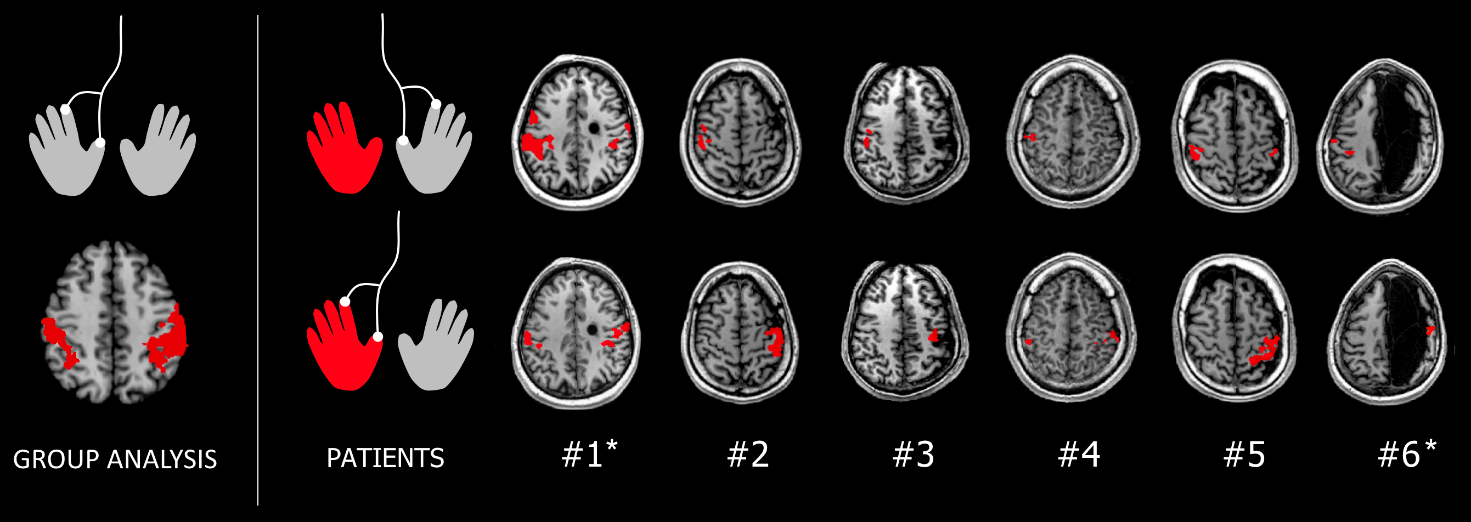
**

Figure 1 represents the activations in primary somatosensory area elicited by the brushing task, for controls group analysis (Group Analysis) and single-subject analysis (Patients). The brushing stimulation in the dominant hand elicits a bilateral S1 activation in the group analysis of controls (S1 localizer). Bilateral representation of S1 was found also in single subject analysis carried on patients. In particular, 2 out of 6 patients had bilateral activations for the brushing stimulation of the dominant hand (grey, top row, patients #1 and #5) and the non-dominant hand (red, bottom row, patients #1, #4). *Right brain lesion
